# A Qualitative Exploration into Experiences and Attitudes Regarding Psychosocial Challenges, Self-compassion, and Mindfulness in a Population of Adults with Cystic Fibrosis

**DOI:** 10.1007/s10880-022-09859-8

**Published:** 2022-02-11

**Authors:** S. Kauser, R. Keyte, M. Mantzios, H. Egan

**Affiliations:** Department of Psychology, Faculty of Business, Law and Social Sciences, Birmingham City University, The Curzon Building, 4 Cardigan St., Birmingham, B4 7BD UK

**Keywords:** Cystic fibrosis, Mindfulness, Self-compassion, Psychosocial challenges, Wellbeing

## Abstract

To investigate the current psychosocial challenges faced by adults with Cystic Fibrosis (CF), while exploring attitudes and experiences of mindfulness and self-compassion. Mindfulness and self-compassion are important resources for supporting psychological and physical well-being, yet there is limited research exploring these positive psychology concepts in CF literature. Twenty UK domiciled adults with a diagnosis of CF, took part in a semi-structured interview. Using a thematic analysis approach, four themes were developed: (a) “I didn’t expect to be here”: Surpassing the CF life expectancy, (b) “Am I psychologically bringing this upon myself?”: Psychological complexities of CF, (c) “I’ve had to really learn to be kind to myself”: The importance of compassion and being self-compassionate, (d) “I think it’s a great tool”: The benefits of practising mindfulness. This research demonstrates a robust need for increased integration of accessible psychological practices into routine CF-care and self-care for adults with CF. Particularly, practises and interventions that draw on the concepts of mindfulness and self-compassion, which may benefit patient’s health and wellbeing profoundly.

## Introduction

Cystic Fibrosis (CF) is a chronic, life-limiting illness caused by defects in the cystic fibrosis transmembrane conductance regulator (CFTR) gene (Cutting, 2015). Once a fatal disease with a median life expectancy of just months in the late 1930s, health outcomes for people with CF have seen remarkable advances with the number of adults living with CF projected to increase by 75% in Western European countries between 2010 and 2025 (Burgel et al., 2015). Following the rapid rise of the adult population living with CF, and despite advances of CFTR modulator therapies, complications in adulthood are increasing creating a mandate to explore novel therapies, clinical care and patient engagement (Bell et al., 2020). In considering the future of CF care, understanding the psychosocial challenges faced in adult life by this relatively new adult CF population, as well as potential strategies to address these challenges is imperative to ensure the optimal CF care and support measures are being offered.

Many challenges faced by adults with CF including education, employment, social support and reproductive health concerns are primary elements of concern to support wellbeing (Muther et al., 2018), indirectly influencing symptom management and treatment adherence (Keyte et al., 2018, 2019a, 2019b, 2020). The psychological distress caused by these new challenges, or by the complexities of daily treatment management of CF, may place individuals at a greater risk for developing poorer mental health or psychological disorders such as anxiety or depression. High rates of depression and anxiety were reported in adolescents and adults with CF, with elevations depicted two to three times higher when compared with community samples (Quittner et al., 2014). Additionally, whilst CFTR modulator therapies provide hope for improving physical health outcomes, several individuals with CF demonstrated worsening depression or anxiety upon commencement of CFTR modulators (Dagenais et al., 2021; Talwalkar et al., 2017). A recent systematic review and meta-analysis further suggested that anxiety and depressive symptoms are common in CF patients worldwide, with a prevalence of 26.22% (95% CI 22.1, 30.2) for anxiety and 12.66% (95% CI 10.6, 14.6) for depression (Guta et al., 2021).

Adults with CF may benefit from psychological interventions on a proactive basis that promote wellbeing. Wellbeing-related research in CF indicate that mental health had a greater impact than physical health in determining several aspects of health-related quality of life, which demonstrates the importance of promoting positive wellbeing (Cronly et al., 2019a). Positive psychology is an applied field which aims to reduce the preoccupation of maladaptive behaviours, negative thinking and mental illness, by instead focussing on building positive individual traits that contribute to the flourishing or optimal functioning of individuals, communities and societies (Seligman & Csikszentmihalyi, 2000). Positive mental health has been associated with better physical health and higher health-related quality of life in a sample of adults with CF, although the authors conclude that future research is needed to explore the potential future benefits of positive psychology interventions across CF populations (Cronly et al., 2019b). Through positive psychology, psychologists can support individuals to develop positive human qualities such as hope, optimism, flow, happiness and gratitude, which buffer against and better prevent mental illness, enabling the ability to flourish (Seligman & Csikszentmihalyi, 2000; Wood et al., 2010). Recent advancements in positive psychology research demonstrate the inclusion and benefits of mindfulness and self-compassion (Egan & Mantzios, 2016; Kotera et al., 2021; Phan et al., 2020; Swickert et al., 2019; Voci et al., 2019).

Mindfulness is described as the ability to focus awareness and attention to the present moment in a non-judgmental way (Kabat-Zinn, 1990). Formal mindfulness programmes include the Mindfulness-based Stress Reduction (MBSR) and Mindfulness-based Cognitive Therapy (MBCT), which are composed of different formal and informal practices, ranging in durations, with shorter practices being as low as 5-minutes (Kabat-Zinn, 1990; Segal et al., 2002; Tan et al., 2014). Brief mindfulness practices are more accessible and enable the formation of a habitual mindfulness practice (Mantzios & Giannou, 2019). Higher levels of mindfulness were predictive of lower psychological distress, lower emotional exhaustion, higher levels of satisfaction and improved quality of life for non-CF samples (Hülsheger et al., 2013; Kaviani et al., 2012; Mantzios et al., 2020; Walach et al., 2006). Mindfulness has been proposed as a potentially important resource for supporting the psychological wellbeing of people with CF (Mantzios & Egan, 2016), and promising findings particularly relate to the use of mindful eating (Egan & Mantzios, 2016; Egan et al., 2021).

Mindfulness can also be discussed as a core element of self-compassion, where the attention, acknowledgement, and acceptance that one is suffering becomes a central aspect of response (Neff & Dahm, 2015). Self-compassion is the ability to demonstrate compassion towards oneself during challenging times, through acts of self-kindness, recognition of common humanity and mindful awareness (Neff, 2011). Recent research in CF shows that quality of life and self-compassion are positively correlated, and each in turn are inversely correlated with negative emotional states and self-criticism (Kauser et al., 2021). Similarly, research outside of CF indicates that higher levels of self-compassion are associated with increased optimism, psychological wellbeing, happiness and life satisfaction, in addition to lower levels of stress, anxiety and depression (Hollis-Walker & Colosimo, 2011; Keyte et al., 2021; MacBeth & Gumley, 2012; Mantzios et al., 2015; Neff et al., 2007; Zessin et al., 2015). Furthermore, a negative relationship between resiliency and mental health was mediated when controlling for self-compassion and mindfulness (Rizal et al., 2020), suggesting that self-compassion and mindfulness can positively influence and explain the relationship between mental health and resiliency (see also Mantzios, 2014). Brief self-compassion practices such as writing compassionate letters demonstrate longitudinal effects of significant increases in happiness for up to six months and significant reductions in depression for up to three months (Shapira & Mongrain, 2010). Additionally, self-compassion interventions such as the Mindful Self-Compassion (MSC) training program, include a combination of interpersonal compassion exercises, guided meditations, and informal home techniques and reported significantly larger increases in self-compassion, mindfulness, and wellbeing which were maintained at six-month, and one-year follow ups (Neff & Germer, 2013). Despite the vast amount of promising data, and propositions of health behaviour change (Egan & Mantzios, 2018; Hussain et al., 2020; Mantzios & Egan, 2017) there has been minimal research exploring the benefits of self-compassion in adult CF populations.

The present research aims to initially explore the current psychosocial challenges faced in adulthood life by this relatively new adult CF population who often face increases in adulthood-related challenges and complications (Bell et al., 2020). A second aim of this research is to understand and explore attitudes and experiences of mindfulness and self-compassion. There remains a paucity of qualitative research focusing on the meaning, experiences, interests, and views of the adult CF population relating to how mindfulness and self-compassion can be used as a beneficial tool to enhance psychological wellbeing. The implementation of flexible and effective interventions around mindfulness and self-compassion constructs could greatly benefit the positive health and wellbeing of the adult CF population.

## Methodology

### Participants

Twenty participants (male: 11, female: 9, mean age: 37 years) were recruited into the study with a mean forced expiratory volume in 1 second (FEV_1_) of 50.43%, and a mean BMI of 23.29 (see Table 1). All participants were English speaking, with a diagnosis of CF, UK domiciled and aged over 18. At one research site 15 patients were approached: 3 refused participation, 2 were withdrawn due to non-completion, and 10 completed the study. At the second research site 18 patients were approached: 2 refused participation, 6 were withdrawn due to non-completion, and 10 completed the study. Reasons for not participating were not recorded. Participants were recruited at two regional Adult CF Centres in the Midlands over a period of four months on pre-arranged days for data collection, using a purposeful sampling method (Creswell & Poth, 2016). Eligible participants were identified by consulting CF outpatient and inpatient lists with CF Consultants to discuss suitability regarding age, capacity, language and health status. Individuals were excluded from the study if they lacked capacity or were non-English speakers. Participants were invited to take part in the study while either attending the centre for a routine outpatient appointment or after inpatient admission to the ward. Data collection was conducted before Kaftrio became available via the National Health Service (NHS) in the UK.

**Table 1:** Participant pseudonym, demographic and medical data

Pseudonym	Sex	Age	Ethnicity	Highest level of education	Employment status	Latest lung function result (FEV_1_)	BMI	CFTR modulator therapy
Usman	M	26	Pakistani	Higher Education	Employed	37%	25.00	No
Chloe	F	49	White	Higher Education	Retired	50%	19.00	Symkevi
Bella	F	35	White	Further Education	Self-employed	n/a	n/a	No
Jasmine	F	21	White	Not applicable	Self-employed	64%	21.80	No
Savannah	F	28	White	Higher Education	Other: Student	43%	21.90	No
Nathan	M	39	White	Higher Education	Employed	52%	23.66	Kalydeco
Aiden	M	22	White	Further Education	Employed	n/a	n/a	No
Arron	M	20	White	Further Education	Unemployed	n/a	n/a	No
Jade	F	50	White	Further Education	Retired	56%	22.02	No
Sabrina	F	41	White	Further Education	Employed	n/a	n/a	Orkambi
Ryan	M	50	White	Higher Education	Unemployed	23%	22.90	Symkevi
Peter	M	52	White	Higher Education	Self-employed	40%	27.40	Kalydeco
Caleb	M	25	White	Higher Education	Employed	30%	21.80	Symkevi
Naomi	F	30	White	Further Education	Employed	83%	21.42	No
Reece	M	39	White	Further Education	Employed	68%	28.58	No
John	M	26	White	Higher Education	Other: Student	81%	25.39	No
Mike	M	55	White	Secondary Education	Retired	38%	n/a	No
Niall	M	38	White	Secondary Education	Unemployed	41%	21.90	Sykmevi
Selena	F	32	White	Further Education	Employed	n/a	n/a	No
Alice	F	52	White	Further Education	Unemployed	n/a	n/a	No

### Semi-structured Interview

Semi-structured interviews explored participants’ experiences and understanding of the psychosocial challenges faced in adulthood, as well as mindfulness and self-compassion. A semi-structured design was used due to the flexibility of the method and use of follow-up questions to gain greater insight into specific discussion points, leading to an enhanced understanding of the topic areas (Turner III, 2010). The psycho-social challenges section explored the impact of CF and treatments, employment and education, eating behaviours, the future and available support which are suggested as key factors in previous literature. The mindfulness and self-compassion sections explored participants’ understanding of the concepts, awareness and experiences of concept-related practices and benefits or drawbacks based on personal experiences.

The interviews were conducted by one researcher and took place either in a private room within the CF Centre (14 participants) or via telephone (6 participants), at the participants discretion. The interviews lasted a mean time of 39.14 min (SD: 12.70) and were recorded using an audio-recording device. Both before and after the interview, the right to refrain from answering questions, to withdraw and to remove personal data were explained. When transcribing, pseudonyms were used to protect participants' identities, and possibly identifiable information was omitted, all participation was voluntary and confidential.

### Ethical Approval

Ethical approval was obtained by the Health Research Authority (HRA) via a NRES Committee (19/NW/0362), the R&D departments at each research site, and by the Universities Ethical Committee.

### Analysis

The interviews were transcribed verbatim by hand, by one researcher. The data were analysed following a contextualist method using Braun and Clarke's (2006) model of thematic analysis, recognising how individuals make meaning of their experiences considering their broader social contexts. The transcripts were manually coded by one researcher, representing important elements of the data, and were subsequently revised and evaluated by everyone on the research team. Codes were grouped together forming themes which were constructed again initially by one researcher. The researchers then evaluated how each code fitted (or not) within the themes, and the codes were confirmed once agreement by everyone on their representation of data were reached, ensuring reliability. The themes were identified in an inductive way to encompass the lived experiences of the CF community, and as such are data-driven rather than being the researcher’s analytic preconceptions.

## Analysis

Thematic analysis identified four themes which were constructed and developed from the data during analysis (see Table 2). Theme one explores some of the current challenges faced in adulthood, and how CFTR modulator therapies are increasing these challenges. Theme two focuses on the psychological wellbeing of adults with CF, including discussions around acceptance, openness, support and identity. Theme three outlines the valuable role of self-compassion on psychological health and wellbeing, contrasted with the negative impact that a lack of compassion and self-compassion can have. Theme four explores participants understanding and experiences of mindfulness, highlighting how mindfulness supports good psychological wellbeing. It is important to note that throughout the themes, there are aspects of individuals’ experiences which do overlap, and this was expected.

**Table 2:** Development from codes to themes

Themes	“I didn’t expect to be here”: Surpassing the CF life expectancy	“Am I psychologically bringing this upon myself?”: Psychological complexities of CF	“I’ve had to really learn to be kind to myself”: The importance of compassion and being self-compassionate	“I think it’s a great tool”: The benefits of practising mindfulness
Codes	The pension process	The impact of physical health fluctuations	Managing personal suffering with self-compassion	Dispositional mindfulness
	Financial worry	Distress, anxiety, depression	Self-care	Psychological health and wellbeing benefits
	Caring for elderly parents	Eating behaviours and weight	Increased patient-centred care	Mindful self-care practices
	CFTR modulator therapies	The ‘invisible’ nature of CF	Lack of compassion from employers	A need for increased mindfulness-based practices
		Isolation and loneliness		

### “I Didn’t Expect to be Here”: Surpassing the CF Life Expectancy

This initial theme demonstrates some of the welcomed challenges faced by a relatively new generation of adults with CF. The term ‘welcomed challenges’ refers to challenges that the CF population are facing today, that occur due to a positive event, in a time of increased life expectancy and a better health status than previous generations of CF patients have experienced.

Several participants, including Mike, vividly remember being informed in childhood that they would not live past their teenage years. The emotional toll of living with a reduced life expectancy and the consequent restrictions imposed upon participants life as a result, was evident across the data. Participants’ narratives indicate a perceived lack of support and understanding regarding the management of adult life with CF, which led to participants feeling unprepared for what was to come. Mike discussed the difficulties of now navigating through adulthood life, and reflected upon how the awareness of a reduced life expectancy impacted his decisions when working in his twenties.

[Mike, 55 years]: “I didn’t expect to be here then…because that’s how they put it into my head when I was younger…I used to think well I don’t need a pension I ain’t gonna be around…will probably affect me now if I do get to retirement age but you know so be it.”

Mike highlights the challenges of an ageing CF population where many adults with CF are now faced with considering important life choices such as pensions, which previous CF generations have not needed to deliberate. This highlights the valuable role of the social worker in a new era of CF care. Mike demonstrates the nature of a welcomed challenge in that the financial implications of not taking out a pension are occurring due to a positive event, an increase in life expectancy.

Despite living with a reduced life expectancy, some participants explained how they have contributed towards a pension but feel the pension process, particularly the state pension is not inclusive of CF needs. Peter like others, contributed towards his pension for 35 years and wanted to access his state pension at an earlier age than the general population due to a deteriorating health status impacting the ability to work.

[Peter, 52 years]: “I still can’t get my pension till 67, same as anybody else who’s healthy…the ability for the older CF population to pull on their state pension earlier even if its reduced, I think would be a great help and I’m sure I’m not alone on that”

Peter proposed the need for increased financial support directed towards the older CF population, and discussed his uncertain financial situation as a widespread challenge. This demonstrates the notion of common humanity, whereby the difficulties associated with the pension process are recognised as part of the shared human experience for the CF population. Peter was able to practice acceptance when recognising his potential future challenges and commented that alongside his wife “together we’ll get there”.

Whilst participants like Peter largely discussed positive and supportive relationships in his life which benefited his psychological wellbeing, some participants mentioned the increasing challenges faced by becoming a carer for one’s parents. Mike discussed the challenges of looking after two elderly parents.

[Mike, 55 years]: “I have to run around for my dad take him to appointments so like if he’s got an appointment at say nine…I have to get up at like six in the morning to get myself ready before I can go over to see him”

Mike explained how his father had a limited understanding about CF believing Mike “sits around watching tv all day”, showing limited compassion towards Mike’s personal needs. Mike prioritises compassion towards his parents over self-compassion. Finding a suitable balance between feelings of not wanting to let one’s parents down and being able to provide all their care needs despite not having capacity, proved particularly challenging.

It is anticipated that this generation of CF patients may experience significant changes in self-identity following the development of CFTR modulator therapies. Some participants noticed both physical and psychological improvements whilst taking Orkambi, Symkevi or Kalydeco, relating to better lung function and consequently daily functioning, such as the ability to walk up the stairs, get dressed and shower, as well as increases in confidence and emotional functioning.

[Peter, 52 years]: “took up paddle boarding…before I never would’ve done that…you know you feel you’re out of breath you’re in trouble so it’s really a lot more energy a lot more confidence to try new things and to me it felt I’d got an extra 10 years”

The ability to engage in more normalised activities due to feeling less breathlessness and more in control following use of Kalydeco for over two years impacted positively upon Peter’s confidence and identity. Caleb also discussed his experiences of taking Symkevi for three months, further highlighting the impact of CFTR therapies on identity in people with CF.

[Caleb, 25 years]: “everyday livings just a lot easier…you sort of know it helps with the cause of the problem rather than just the symptoms…so you’re actually er you’re feeling a bit more normal…which is all you can ask for”

Caleb highlights that because of Symkevi he was able to achieve a sense of normalcy, and therefore adopt a ‘normal’ identity. The licensing of CFTR therapies encouraged a sense of hopefulness to the participants, alongside a general consensus regarding the importance for the next generation of people with CF to plan for their future as they are more likely to live longer into adulthood.

Overall, the welcomed challenges discussed mainly refer to the financial implications of the pension process and the changes in relationship dynamics with one’s parents. In light of CFTR therapies and with the extended life expectancy, it is important to note that patients, clinicians and the wider society are currently re-learning what living with CF means today. This theme demonstrates the need for increased awareness of such challenges to be integrated into CF care, as well as the provision of adequate support measures to help individuals accept the changes and challenges faced in a relatively new era of adulthood life with CF. The difficulties of managing CF-related challenges and more specifically the psychological burden caused by such challenges are explored more fully in the second theme.

### “Am I Psychologically Bringing this Upon Myself?”: Psychological Complexities of CF

This theme initially focuses on the difficulties of coping with the complexities of CF and the negative impact this had on participants psychological wellbeing. Data shows that everyday life activities such as working and eating, can become difficult to manage especially alongside one’s personal and social life, thus increasing distress. Niall discussed how sometimes health-related CF fluctuations can cause a detrimental cycle of repercussions affecting his emotional wellbeing and impacting on his motivation to comply with treatment adherence.

[Niall, 38 years]: “Then when something bad happens…start to feel miserable it tends to have a knock-on effect you could cause on your treatment then you feel worse, and you have like a don’t give a shit attitude”

Here Niall demonstrates a lack of kindness or self-compassion, being judgemental rather than accepting of his suffering, resulting in a state of decreased psychological wellbeing and an avoidant stance of not caring.

Many participants were able to clearly describe how increased levels of distress caused by CF, manifested in increased levels of anxiety or depressive symptoms, this was especially evident with Nathan and Jade. Jade identified the impact of having multiple serious CF-related health complications during adulthood.

[Jade, 50 years]: “There’s so many things that go wrong with CF and I was thinking god, is it me? Am I psychologically bringing this upon myself? Am I creating these and then they’re manifesting themselves? So I found that hard”

Jade discussed being very in tune with both her mind and body, and felt confident to distinguish when something was wrong with her health. The idea that her symptoms might be psychosomatic diminished that confidence causing considerable psychological distress. Although Jade highlighted the overall excellence of CF clinical teams, on many occasions she felt dismissed and described having to “battle” for the correct clinical care. Jade was eventually diagnosed with a (redacted) tumour after experiencing and persistently reporting numerous symptoms over a two-year period. These experiences of missed diagnoses negatively affected Jade’s psychological wellbeing, she described changing from being “very pragmatic and brilliant in an emergency” to feeling “very stressed very quickly”, and repeatedly questioned the rationality of her thoughts and experiences around her own health.

Several participants described challenges around the complexities of eating and weight. Ryan, Mike and Sabrina discussed the physical constraints of cooking: difficulty bending down to reach the freezer, lifting heavy pans, breathlessness from cooking steam, needing to use a stool to sit down when cooking. This reduced participants ability and desire to cook and negatively added to the already existing complexity of constant awareness and pressure to consume a high calorie intake diet, as mentioned by Niall.

[Niall, 38 years]: “in the same breath I’m trying to keep the calories up so it’s hard, if you was just to eat healthy but keep calories on that would be hard…so it’s just like er merry-go-round at the moment of what’s good and what’s right”

Niall identifies the difficulty and confusion in finding an adequate balance between eating what he perceives as good for this body (healthy) or what is right for his CF (high calorie foods). Many participants described heightened disease-related distress when self-regulatory efforts to achieve adequate calorie intake did not successfully translate into weight gain in the desired places. A reoccurring concept of weight disproportion resulting in a bigger stomach with skinny arms and legs was common and was described as a distinctive feature for people with CF. This caused body-consciousness and provides an understanding of how CF can impact identity in adults with CF. Participants found it difficult to balance the idealistic weight of a healthy BMI encouraged by clinicians to ensure a good health status for their CF, with their personal idealistic view of how they would like to look.

Several participants, such as Selena, discussed suffering with CF-related challenges in silence due to feeling misunderstood and not wanting to burden others, which further impacted their psychological wellbeing.

[Selena, 32 years]: “She says you know there’s not a chance you can go [for a walk]?…it’s boring just sat in the house…I wanted to scream at her but then I think because I don’t share how tired and...unwell I am…she doesn’t realise that I’m not just saying no because I just don’t wanna go for a walk, I’m saying no cus I can’t go for a walk”

Coping with the physically ‘invisible’ nature of CF was challenging for Selena, as she felt that her physical limitations were often unseen or overlooked by others. Selena found difficulty communicating her needs behaviourally (i.e., by taking a rest), or verbally (i.e., to others), to make them visible, which reduced opportunities for others to respond compassionately to these needs. This reluctance with verbalising the limitations caused by CF was echoed by others, the main reason was not wishing to be a burden. Like Mike in the prior theme, Selena prioritises demonstrating compassion towards her parents, at the expense of self-compassion, which increased feelings of pressure and guilt about not having the physical energy or capacity to complete everyday tasks. Whilst Selena valued the invisible nature of CF as it allowed her to adopt a normal identity, it was evident that the desire to portray a sense of normalcy reduced the opportunities of receiving support from others.

Feelings of isolation and loneliness in dealing with one’s CF and CF-related complications were evident across many interviews. Chloe and Caleb spoke of keeping their concerns to themselves for many different reasons, not least of which was to avoid feeling like a burden.

[Chloe, 49 years]: “I don’t really confide in my mum about how I’m doing…she’s 84 she shouldn’t worry, I’m ok you know…it is quite difficult cus there’s not really anyone that really gets it, that you can talk to, because it’s kind of its such a tough conversation to have with somebody erm and you don’t want to burden them”

The fear of burdening loved ones with her own suffering, evidently shapes the way Chloe copes with her CF challenges and this is demonstrable through the contrasting feelings of “I’m ok” to admitting a few words later that actually “it is quite difficult”.

Several participants discussed how advancements in technology and social media provided a supportive tool for coping with CF. Savannah acknowledged how online gaming sessions and Twitter increase opportunities for individuals with CF to interact with others if they wish to do so. Despite an awareness of these benefits, Savannah chose not to use social media for CF support as she personally finds it not to be a positive experience.

[Savannah, 28 years]: “I have a few people that I follow on Instagram but sometimes it’s so depressing…erm I literally unfollow it”

[Arron, 20 years]: “It [Twitter and Facebook] really can help you out cus you read somebody’s experience and think wow that’s actually very close to mine what can I pull out of that into mine”

For participants who use social media as an escape, the data suggested these participants do not want to be reminded of their CF, especially when others are suffering with reduced health as it could serve as a reminder of how their own health could deteriorate one day, causing a negative impact upon psychological wellbeing. However, Bella and Arron described the benefits of Twitter and Facebook as a platform to learn from others and share one’s own CF experiences. Bella discussed how she was part of a CF group where members were advised not to speak about CF but instead more general topics such as music or movies which proved beneficial for her wellbeing.

Participants who demonstrated openness and acceptance about CF with others, often described better psychological wellbeing. For example, Arron was very open about his challenges and discussed how talking to others about CF improves peoples understanding and awareness of his needs.

[Arron, 20 years]: “My [sports] club…everybody up there is absolutely fantastic everybody’s aware of what my problems are, everybody’s aware of my CF…they know if I need to sit down take a break…nobody cares…everybody’s sound”

Arron discussed how accepting his illness identity allows him to be comfortable and open about his CF needs, which in turn enables others to be accepting of his needs and to offer appropriate, compassionate support.

Overall, this theme identified in what ways adults with CF are still struggling to cope with the complex nature of the condition and the detrimental impact that this is having on psychological wellbeing. High levels of stress or isolation with CF challenges is not only detrimental to psychological health, but also negatively impacts on physical health, quality of life and identity. The value that compassion and self-compassion can have on psychological wellbeing was evident, and will be discussed in the following theme.

### “I’ve Had to Really Learn to be Kind to Myself”: The Importance of Compassion and Being Self-compassionate

This theme explores the beneficial role that self-compassion has in enhancing psychological health and wellbeing, alongside the negative impact resulting from a lack of compassion and self-compassion. During physical health fluctuations, some participants like Jade, demonstrated self-compassion through increased levels of understanding and care towards personal suffering.

[Jade, 50 years]: “I woke up really poorly and there was just no way I could keep [diabetic appointment] so I didn’t, I rang and then just stayed in bed because erm I needed to do what was best for me that morning and I needed to sleep, whereas [participant’s name] 10 years ago would’ve probably put myself in hospital trying to keep the appointment”

The balance between attending to the medical needs of CF alongside non-CF medical needs, and more holistic self-care needs was difficult for many. Jade described how in the past, her desire to be a compliant CF patient took precedence over her self-care needs, which sometimes resulted in poor health outcomes. Several participants discussed that they have had to learn to be kind to themselves and put themselves first. For Jade, this recent acceptance of managing suffering with sympathy and kindness has resulted in greater psychological wellbeing.

The importance of self-care practices was apparent throughout the data, especially during challenging and stressful situations. Self-care was enacted in many ways, from time spent doing enjoyable activities such as listening to music, going shopping, going for drives, to more health-related behaviours such as getting an early night, using the gym, and taking a walk. Many participants talked about the difficulties in finding time to practice self-care, for some, time in hospital provided a much-needed opportunity to be more self-compassionate and mindful.

[Jasmine, 21 years]: “When I come into hospital it’s like everything just comes to a standstill and you have no choice but to think of what’s going on in your life…like this is the reality of your life and you need to take time out now to actually look after yourself”

[Sabrina, 41 years]: “the only time I do [take time out for myself] is when I’m in hospital”

Hospitalisation is often due to health deteriorations or exacerbations and as such is experienced as a negative health and life event. Jasmine, Sabrina, and others exemplify a way of responding to their present moment suffering with self-kindness and acceptance. They reframe hospital admittance from a negative event to a means of allowing them to take this much-needed break and to prioritise their wellbeing. This also denotes how difficult they find this in everyday life.

There was a common discussion regarding increased patient-centred care and compassion within CF clinical care over time, during hospital admittance but also during outpatient appointments. Some of the oldest participants including Peter, Chloe and Jade discussed their experiences and appreciation of feeling more involved in their care.

[Peter, 52] “I noticed a shift in CF care…it used to be you’d sit there, and the doctors would discuss you with each other and then tell you…within the last decade suddenly you became very involved as a patient you were trusted to get the medical information they would tell you a lot more about where you’re at and your treatment…you felt that you were more empowered around your health”

Prior feelings of nervousness, lack of control, and worry about receiving CF care had changed into feeling more empowered, demonstrating the importance of compassion on psycho-emotional wellbeing and inner development (and personal growth), by clinical CF teams through use of a more patient-centred approach. Jasmine reflects the current consensus about the CF clinical teams, with many participants referring to them as a second family. Generally, participants felt that CF clinical teams demonstrated increased compassion over time, and this was particularly appreciated when other aspects of life were difficult. Issues of managing CF and paid employment were common.

Several participants felt that they were unable to be self-compassionate whilst at work and that they did not receive compassion from their employers. Savannah highlights how working in a managerial role had detrimentally impacted her health.

[Savannah, 28 years]: “They were pushing me when I wasn’t able to be pushed…definitely impacted…my mental health as well as like my physical health…it was so stressful cus you’re just kinda tryna keep everything ticking over and if someone isn’t supportive of you taking time off you push yourself even harder when maybe I needed to kind of calm down”

Evidently, Savannah was aware of a need for more self-compassionate behaviour however due to external factors at work and a lack of compassion from employers, engaging in positive self-kindness was not possible. Instead, Savannah found it harder to take the time needed to adhere to her treatment regime and she increased her intake of alcohol as a means of coping with work-related stress. Other participants shared similar experiences of using alcohol and illicit drugs to cope with distress. Additionally, Savannah confirmed the impact of this stressful period during employment, on her mental health, where she isolated herself from others, causing excessive strain on her social life.

Overall, self-compassion and compassion from others are important factors which impacted upon participants experiences of psychological health and wellbeing. The recognition of importance around self-compassion, but lack of implementation, demonstrates a need for the development of more ways to effectively practice self-compassion and allow for prioritisation of self-care for people with CF. Additionally, the need for employers to demonstrate a kinder and more compassionate approach towards people with CF in the workplace is vital, alongside the need for those with CF to find ways of coping with work demands and stress in a more self-compassionate manner. Self-compassion and mindfulness go hand in hand, in that we cannot ignore one’s own suffering and feel compassion for it at the same time, both concepts are equally important in ensuring good psychological wellbeing. The next section will focus on discussions around mindfulness.

### “I Think It’s a Great Tool”: The Benefits of Practising Mindfulness

The final theme explores ideas around mindfulness, examining how the key concepts of mindfulness can enable good psychological wellbeing. Mindfulness was interpreted differently by participants, often discussed alongside tenets of trait mindfulness, with additional reference to self-help measures. Despite several participants claiming no initial understanding of mindfulness, many of these people indirectly discussed approaching CF-related challenges with a mindful attitude through the topics of present moment awareness, acceptance, gratitude, and mind–body connection. When participants responded to suffering with a mindful awareness of negative thoughts and emotions, challenges were approached without judgement, avoidance, or repression. Selena and others highlighted the importance of approaching life with a mindful awareness, due to the unforeseeable nature of CF health fluctuations. Largely Selena’s understanding of mindfulness, like many others, derived from the teachings of a clinical psychologist.

[Selena, 32 years]: “you have to live for now…because having CF you learn that each day is special…so you get the opportunity to do something grab it with both hands because you might not be able to do it again…it’s the thought you don’t know…what’s gonna happen, I’ve had infections where I was fine one day and the next I was wiped out, my lung function dropped and I was on oxygen”

Selena demonstrated the significance of dispositional mindfulness by reflecting on self-regulation of attention to the present moment. Living in the present moment facilitated Selena’s ability to enjoy everyday life through open and non-judgmental orientation to experiences, as well as associations with gratitude. Many participants described gratitude in being appreciative of their health, thus indirectly exploring concepts of mindfulness. Despite little use of the term ‘mindfulness’, mindfulness became a way of life for participants, especially when describing adulthood experiences with CF.

For John and others, the term mindfulness was a familiar concept that was perceived as a stress-relief, a mental-reset, a means to manage and clarify thoughts and a tool to energise oneself, facilitate anxiety or reduce worry. John was introduced to mindfulness in paediatric care, which involved taking short periods of time out during the day when feeling overwhelmed.

[John, 26 years]: “taking a quiet 5 min out and just taking time to just focus on your body, feel the breathing, feel muscles in your legs…just a bit of a mental reset really”

John explains how he uses mindfulness to experience thoughts, feelings, and sensations as they happen in the present moment, whilst also demonstrating an understanding of the mind–body connection, an important characteristic of mindfulness. John describes the mind–body process of reconnecting with the body, by focussing on his breath and experiencing the sensations felt throughout the body. Incorporating holistic self-care through equally attending to one’s body and mind, using the notions of mindfulness, is both necessary and important in facilitating behavioural change and improving both psychological and physiological wellbeing. The regular use of mindful self-care practices over time, resulting in John’s acquirement of state mindfulness, led to increases in trait mindfulness which was evident throughout discussions demonstrating an open and non-judgmental orientation to experiences, leading towards a more mindful and less distressed disposition. A notable aspect of John’s interview reflecting the acquirement of heightened levels of trait mindfulness relate to his ability of perceiving challenging situations as a “phase”, as opposed to over identifying with these situations. John was therefore able to consciously step back from stressful encounters for example, when approaching assessment deadlines.

Some participants practised mindfulness through meditation and yoga, either alone or in a group setting, via self-care measures including mindfulness-based audio recordings and through applications such as Headspace. An interest towards mindfulness was demonstrated by most participants, with reference to partaking in future mindfulness practices, and some resuming with prior practices. For example, Reece discussed conducting internet-based research into mindfulness concepts, particularly present moment awareness, which he has been actively trying to implement into daily life. Peter engaged with meditation practice for over 20 years, yet discussed how there is minimal information and direction towards mindfulness practices for individuals with CF.

[Peter, 52 years]: “Certainly things like meditation…should be offered up more to patients from an earlier age…whether it’s in annual reviews…the opportunity to go on this meditation course or mindfulness…it’s a great tool, I haven’t seen that offered up anywhere…I’ve heard it talked about as a benefit but in a practical way I don’t think it costs a lot, I think it can have real practical benefits”

Despite the demonstrable interest and clear psychological health and wellbeing benefits of mindfulness, Peter highlights a robust need for increased proactive mindfulness-based tools offered to CF patients. Generally, those participants who engaged with mindfulness or demonstrated mindfulness within their lives were less likely to engage with maladaptive coping mechanisms such as health risk behaviours. This theme also highlights the pivotal role that psychologists and clinical teams play in increasing the awareness and practise of mindfulness, supporting patients’ psychological wellbeing through self-care practices or clinical intervention. Overall, the data shows how increased support measures around mindfulness and self-compassion could be enhanced to help individuals manage CF-related challenges and psychological burden, leading to a better quality of life.

## Discussion

The results initially demonstrate how this relatively new era of adulthood life with CF can present several welcomed challenges, particularly how these challenges can negatively impact one’s psychological wellbeing when accessibility and provision of support measures are limited. The latter half of the results explored participants’ views and experiences around mindfulness and self-compassion, demonstrating the benefits and need for increased psychological intervention to enhance health and wellbeing.

Data suggested that the adult CF population demonstrate a lack of preparedness for adulthood life with CF. Many individuals are grateful for continuously exceeding the estimated CF life expectancy, but also worried for what lies ahead, especially the financial implications. These findings support Bell et al. (2020), highlighting how CF centres must ensure adequate steps are taken to meet the needs of ageing CF patients. Advancements of modulator therapies are likely to continue increasing life expectancy, and the current study demonstrates how the disease also continues to present challenging psychosocial complexities, negatively impacting quality of life in adulthood. Participants discussed psychosocial challenges, which were unique to the nature of CF, and others that were more generally aligned with many chronic conditions, which corroborates with prior literature discussing the wide-ranging scope of adulthood CF challenges (Muther et al., 2018).

With anxiety and depression symptoms increasing following the use of CFTR modulator therapies (Dagenais et al., 2021), it is important to consider the continuous implications of health changes. The present results demonstrated how out of tune individuals felt with their mind and body when trying to understand their health, something to be cautious of when considering the implications of CFTR modulator therapies on patients’ health, both physical (i.e., difficulties when detecting health fluctuations) and emotional (i.e., not feeling in control). The current findings also emphasised issues around weight disproportion, body-consciousness, and the consequential impact on one’s identity, signifying the need for clinical interventions alongside modulator therapies, where nutritional and emotional counselling may create opportunities for patients to adapt more easily to changes.

This study demonstrates that adults with CF require further emotional support measures to help cope with the complexities of adulthood life, especially during health-related CF fluctuations. Results indicated that people often became overwhelmed during ill health, seeing an increased use of unhelpful coping mechanisms such as isolation and self-criticism. When participants were unable to mindfully cope with their suffering, for example through a lack of acceptance, they also experienced increased feelings of guilt, pressure, and distress. It was apparent how self-compassion intertwined with mindfulness, especially when relating to acceptance, demonstrating a dynamic process leading to enhanced wellbeing (Neff & Dahm, 2015). Introducing brief mindfulness- and compassion-based interventions proactively could increase over time trait mindfulness (e.g., Kiken et al., 2015), and enhance a disposition that could help buffer against reductions in wellbeing for adults with CF.

The present findings discussed the benefits of mindfulness in relation to improved psychological wellbeing, and further highlighted a demonstrable interest in the concept. Kaviani et al. (2012) demonstrated the effectiveness of mindfulness practice in improving quality of life, and reducing anxiety and depressive feelings. Similarly, this study demonstrated through descriptive participant experiences how mindfulness has been, and could be used to improve health and wellbeing within the adult CF population. However, participants also discussed a lack of information and accessibility of mindfulness practices for CF patients, demonstrating a robust need for increased pro-active mindfulness-based tools offered to CF populations. Through the current findings, mindfulness can be seen to enable a more open and non-judgmental orientation to challenging experiences, providing a self-care experience that becomes more bearable through present moment attention as opposed to overidentifying with one’s negative thoughts and feelings. With this knowledge, psychological interventions that enhance the ability to focus awareness and attention to the present moment in a non-judgmental way, such as brief mindfulness practices (Mantzios & Giannou, 2019), could be created and made more accessible to the adult CF population on a proactive basis.

Importantly, the presence of suffering within CF is an element that requires a deeper orientation and response that is better experienced through self-compassion, where an understanding, kind and collective self-perception can strengthen identity and tolerance of suffering moments. Furthermore, mindfulness and self-compassion interventions may need a more intense reflection on the body, versus only the cognitive and emotional instigations that are seen in mainstream literature. In support with prior research, the present results indicate that physiological problems and the associated suffering, a body-mind continuum, requires more of a body-mind approach of those interventions (Egan & Mantzios, 2018; Hussain et al., 2020; Mantzios & Egan, 2017). Through effective integration of mindfulness practices which also work to enhance one’s self-compassion into existing medical and psychological CF care, patient’s health and wellbeing may benefit profoundly (see also Mantzios & Egan, 2016).

Additionally, the concept of gratitude and it’s interlink with mindfulness was evident within the data, often demonstrated through participants habitual awareness and appreciation of their health and positive aspects of life. Discussions around dispositional mindfulness highlighted how self-regulating attention to the present moment allowed participants to enjoy everyday life through a non-judgmental awareness of experiences that brought about happiness and gratefulness. Like prior research, the present study highlighted beneficial associations between gratitude, mindfulness, and wellbeing (Swickert et al., 2019; Voci et al., 2019; Wood et al., 2010). Whether gratitude can play an intervening role remains a question for future research, but the potential of working side by side in a more direct way may propose a specialised care plan for people with CF.

There are three limitations to the present study. First, 19 participants were from a white ethnic background, and only one individual identified as Pakistani. Then again, the CF demographic mainly consists of a Caucasian background, making the present sample representative. Second, the study lacked adequate data regarding mental health diagnosis of anxiety or depression, which may have provided more insight into experiences of concepts such as mindfulness and self-compassion. Third, it is important to consider that data collection was conducted prior to the availability of Kaftrio via the NHS, which may relate to experiences of psychological distress exacerbated upon the commencement (or not) of modulators (Dagenais et al., 2021). Future research should continue to explore wellbeing and the potential of mindfulness, self-compassion and gratitude in a population that is shifting into differing eras of health, and potential implications to identity and wellbeing.

Overall, this research explores the current psychosocial challenges faced by adults with CF whilst also examining how mindfulness and self-compassion are viewed and experienced by this population. Alongside the scientific advancements of CFTR modulator therapies, proactive psychological interventions which incorporate mindfulness practice may assist CF teams with enhancing positive mental health, coping skills and resiliency in adults with CF whilst also forming an important preventative measure of mental health. The findings establish a way forward for CF care through demonstrating a necessity for increased proactive psychological intervention and ongoing education, accessibility, and provision of brief mindfulness and self-compassion interventions for adults with CF.

## Data Availability

The data that support the findings of this study are available on request from the corresponding author.
